# Immune marker levels in severe mental disorders: associations with polygenic risk scores of related mental phenotypes and psoriasis

**DOI:** 10.1038/s41398-022-01811-6

**Published:** 2022-01-26

**Authors:** Maren Caroline Frogner Werner, Katrine Verena Wirgenes, Alexey Shadrin, Synve Hoffart Lunding, Linn Rødevand, Gabriela Hjell, Monica Bettina Elkjær Greenwood Ormerod, Marit Haram, Ingrid Agartz, Srdjan Djurovic, Ingrid Melle, Pål Aukrust, Thor Ueland, Ole Andreas Andreassen, Nils Eiel Steen

**Affiliations:** 1 NORMENT, Division of Mental Health and Addiction, Oslo University Hospital & Institute of Clinical Medicine, University of Oslo, Oslo, Norway; 2 Department of Medical Genetics, Oslo University Hospital, Oslo, Norway; 3 Department of Psychiatry, Ostfold Hospital, Graalum, Norway; 4 NORMENT, Institute of Clinical Medicine, University of Oslo, Oslo, Norway; 5 Department of Psychiatric Research, Diakonhjemmet Hospital, Oslo, Norway; 6 Department of Clinical Neuroscience, Centre for Psychiatric Research, Karolinska Institutet, Stockholm, Sweden; 7 NORMENT, Department of Clinical Science, University of Bergen, Bergen, Norway; 8 Research Institute of Internal Medicine, Oslo University Hospital, Rikshospitalet, Oslo, Norway; 9 Faculty of Medicine, University of Oslo, Oslo, Norway; 10 Section of Clinical Immunology and Infectious Diseases, Oslo University Hospital Rikshospitalet, Oslo, Norway; 11 K.G. Jebsen - Thrombosis Research and Expertise Center (TREC), University of Tromsø, Tromsø, Norway

**Keywords:** Schizophrenia, Bipolar disorder

## Abstract

Several lines of evidence implicate immune abnormalities in the pathophysiology of severe mental disorders (SMD) and comorbid mental disorders. Here, we use the data from genome-wide association studies (GWAS) of autoimmune diseases and mental phenotypes associated with SMD to disentangle genetic susceptibilities of immune abnormalities in SMD. We included 1004 patients with SMD and 947 healthy controls (HC) and measured plasma levels of IL-1Ra, sIL-2R, gp130, sTNFR-1, IL-18, APRIL, and ICAM-1. Polygenic risk scores (PRS) of six autoimmune disorders, CRP, and 10 SMD-related mental phenotypes were calculated from GWAS. General linear models were applied to assess the association of PRS with immune marker abnormalities. We found negative associations between PRS of educational attainment and IL-1Ra (*P* = 0.01) and IL-18 (*P* = 0.01). There were nominal positive associations between PRS of psoriasis and sgp130 (*P* = 0.02) and PRS of anxiety and IL-18 (*P* = 0.03), and nominal negative associations between PRS of anxiety and sIL-2R (*P* = 0.02) and PRS of educational attainment and sIL-2R (*P* = 0.03). Associations explained minor amounts of the immune marker plasma-level difference between SMD and HC. Different PRS and immune marker associations in the SMD group compared to HC were shown for PRS of extraversion and IL-1Ra ([interaction effect (IE), *P* = 0.002), and nominally for PRS of openness and IL-1Ra (IE, *P* = 0.02) and sTNFR-1 (IE, *P* = 0.04). Our findings indicate polygenic susceptibilities to immune abnormalities in SMD involving genetic overlap with SMD-related mental phenotypes and psoriasis. Associations might suggest immune genetic factors of SMD subgroups characterized by autoimmune or specific mental features.

## Introduction

Bipolar disorder (BD) and schizophrenia (SCZ) are among the leading causes of disability worldwide. The pathophysiology of these severe mental disorders (SMD) is not fully understood. Their estimated heritability is 60–80% and recent genome-wide association studies (GWASs) have revealed a large number of common genetic risk variants [1, 2] with extensive overlap between the disorders [3]. Moreover, evidence suggests extensive genetic overlap with several associated traits [4] and comorbid disorders [1, 5]. The growing body of GWAS can be used to generate polygenetic risk scores (PRS) of SMD-related phenotypes to study core features of SMD [6].

Involvement of the immune system in SMD is supported by several lines of evidence [7, 8]. Genetic loci in immune-related regions are associated with SCZ and BD [9] and infections during pregnancy, potentially inducing immune activation, increase the child’s risk of developing SCZ and BD decades later [10]. Similarly, large registry studies have identified an increased risk associated with prior infections [11, 12]. Inflammation seems to modulate brain mechanisms underlying clinical characteristics of SMD [13]. Moreover, similar signs of systemic low-grade immune activation and inflammation are found in SCZ and BD [14] and associations with symptoms [15] and pharmacological treatment response [16] are reported. However, the causative mechanisms of immune abnormalities, as well as the pathogenic importance of the different pathways that are upregulated, are unknown.

Autoimmune disease is characterized by immune-mediated attacks by autoantibodies and self-reactive lymphocytes in particular T cells and is a known comorbidity of SMD [17]. There is evidence of shared genetic risk with SMD [9] and neuropsychiatric symptoms in autoimmune diseases [17]. While the immune signaling system is extensive and autoimmune diseases are associated with different circulating immune markers [18], some of these markers are also linked to SMD, suggesting not only shared genetic risk factors, but also shared immune-related mechanisms. Such markers include interleukin-18 (IL-18) and interleukin-1 receptor antagonist (IL-1Ra) implicated in among others rheumatoid arthritis (RA), a proliferating-inducing ligand (APRIL) involved in RA and systemic lupus erythematosus (SLE), interleukin-6 (IL-6) in RA and inflammatory bowel disease (IBD) and soluble interleukin-2 receptor (sIL-2R) as a marker of T-cell activation involved in a wide range of autoimmune disorders like IBD and SLE [19–22].

Several mental traits and disorders are genetically associated with SMD [23]. Such examples of mental disorders include major depressive disorder (MDD), anxiety disorders, and autism spectrum disorders (ASD) [24–27]. Further, the genetic association between SMD and different mental traits include cognitive abilities [28], a core impairment of SMD and personality traits of openness, extraversion, and neuroticism [29, 30]. The immune system is also suggested to be involved in these SMD-related disorders and traits. Comorbid mental disorders are associated with immune marker abnormalities [31–34] and immune-related genetic factors [9, 35, 36]. Cognitive abilities correlate with immune factors [37] and immune mechanisms in personality traits are indicated [38].

Physiological levels of immune markers are in addition to a range of non-genetic factors, influenced by complex genetics [39]. Thus, mapping the genetic architecture of immune abnormalities in SMD may provide novel pathophysiological knowledge. Applying multiple PRS of SMD-related phenotypes for explaining characteristics within SMD was recently suggested [4]. Here, we tested the hypothesis that PRS of autoimmune or SMD-related mental phenotypes with immune associations can explain detectable, but small amounts [40] of immune marker abnormalities in SMD. We applied PRS of autoimmune diseases and mental phenotypes, including mental disorders [31–33, 41], cognitive phenotypes [37, 42], and personality traits [38] in a naturalistic sample of SMD to investigate associations with immune markers. SCZ and BD spectrum-specific analyses were further explored.

## Methods

### Study setting

The study is a part of the Thematically Organized Psychosis Study (TOP) at the Norwegian Centre for Mental Disorders Research (NORMENT). The TOP study includes patients from the major hospitals in the Oslo region with a diagnosis of a SCZ spectrum (schizophrenia, schizophreniform disorder, schizoaffective disorder, delusional disorder, brief psychotic disorder, and psychosis not otherwise specified (NOS), from here termed “SCZ”) or BD spectrum (bipolar 1 disorder, bipolar 2 disorder, bipolar disorder NOS, and major depressive disorder with psychotic features, from here termed “BD”) disorder as the main inclusion criteria. Participants must be able to provide written informed consent and be between the ages of 18–65. Healthy controls (HC) are randomly selected from statistical records from the same catchment areas as the patients. HC are included based on no history of severe mental illness, illicit drug abuse or dependency, or close relatives with severe mental illness. Both patients and HC are excluded based on somatic conditions interfering with brain function, neurological disorder, IQ < 70, or history of severe head trauma. In this study, we additionally applied the following exclusion criteria: data of participants other than European ethnicity were not included in the analyses to avoid issues with population stratification, and participants with CRP level of 10 or above (*N* = 114) were excluded to remove potential effects of acute infection on the immune markers. The resulting sample with immune measures included *N* = 1004 patients with SMD and *N* = 947 HCs (Table 1), out of which PRS data was available for *N* = 1802 individuals (Table 2). Immune measures from the current sample have been reported in overlapping samples [43–47].

**Table 1:** Demographics and clinical data.

	SCZ, *N* = 595	BD, *N* = 409	HC, *N* = 947	SMD vs HC^a^	Subgroup comparisons^b^
Sex female, *N* (%)	253 (42.5)	240 (58.7)	436 (46.0)	ns	HC, BD > SCZ
Age, mean (SD)	31.6 (10.1)	35.0 (12.4)	34.0 (9.3)	0.04	HC, BD > SCZ
GAF-symptoms, mean (SD)	45 (12)	58 (12)		n/a	BD > SCZ
GAF-function, mean (SD)	46 (13)	56 (13)		n/a	BD > SCZ
Medication, *N* (%)					
Antipsychotics	494 (83.0)	200 (48.9)		n/a	SCZ > BD
Anticonvulsants	82 (13.8)	147 (35.9)		n/a	BD > SCZ
Lithium	15 (2.5)	69 (16.9)		n/a	BD > SCZ
Antidepressants	170 (28.6)	147 (35.9)		n/a	BD > SCZ
Diagnose, *N* (%)					
Schizophrenia	339 (57.0)			n/a	n/a
Schizophreniform	36 (6.1)			n/a	n/a
Schizoaffective	85 (14.3)			n/a	n/a
Bipolar disorder 1		229 (56.0)		n/a	n/a
Bipolar disorder 2		123 (30.1)		n/a	n/a
Bipolar disorder NOS		19 (4.6)		n/a	n/a
Major depressive disorder		38 (9.3)		n/a	n/a
Psychosis NOS	82 (13.8)			n/a	n/a
Brief psychotic disorder	14 (2.4)			n/a	n/a
Delusional disorder	39 (6.6)			n/a	n/a

*BD* bipolar spectrum disorders, *GAF* general assessment of functioning scale, *ns* not significant, *n/a* not applicable, *SCZ* schizophrenia spectrum disorders, *SMD* severe mental disorders.

^a^*P* value of SMD versus HC: chi square for categorical variables, *t* test for continuous variables, ^b^chi square for categorical variables, ANOVA for continuous variables.

**Table 2:** Polygenic risk scores.

Polygenic risk scores^a^, mean (SD)	SCZ	BD	HC	*P* value^b^	SMD vs HC
Autoimmune diseases and CRP					
CD	0.04 (1.0)	0.03 (0.9)	−0.07 (1.0)	0.18	n/a
IBD	0.01 (1.0)	−0.04 (1.0)	0.05 (1.0)	0.13	n/a
PSOR	−0.03 (0.9)	−0.04 (0.9)	0.08 (0.9)	0.06	HC > SMD
RA	−0.002 (1.0)	−0.04 (1.0)	−0.02 (1.0)	1.0	n/a
SLE	−0.03 (0.8)	−0.05 (0.7)	−0.12 (0.7)	0.74	n/a
T1D	0.05 (0.8)	0.10 (0.8)	0.06 (0.8)	0.12	n/a
CRP	−0.02 (0.9)	0.00 (0.9)	−0.04 (0.9)	0.84	n/a
Mental disorders					
ADHD	0.05 (1.0)	0.11 (1.0)	−0.09 (0.9)	<0.001	SMD > HC
ANX	0.17 (1.0)	0.12 (0.9)	−0.09 (1.0)	<0.001	SMD > HC
ASD	0.03 (1.1)	0.08 (1.1)	−0.01 (1.0)	0.15	n/a
MDD	0.06 (1.0)	0.01 (1.0)	−0.08 (1.0)	0.04	SMD > HC
PTSD	0.05 (1.0)	−0.02 (0.9)	−0.11 (0.9)	0.002	SMD > HC
Cognitive traits					
COG	−0.08 (1.0)	0.02 (1.0)	0.07 (1.0)	0.02	HC > SMD
EA	0.11 (1.0)	0.08 (1.0)	0.17 (1.0)	0.10	HC > SMD
Personality traits					
EXTRA	0.04 (1.0)	0.05 (1.0)	−0.02 (1.0)	0.16	n/a
NEURO	0.01 (1.0)	−0.04 (1.0)	−0.08 (1.0)	0.16	n/a
OPEN	0.10 (1.0)	0.03 (1.0)	−0.01 (1.0)	0.10	SMD > HC

*ADHD* attention-deficit hyperactivity disorder, *ANX* anxiety, *ASD* autism spectrum disorder, *BD* bipolar spectrum disorders, *CD* Crohn’s disease, *COG* cognition, *CRP* C-reactive protein, *EA* educational attainment, *EXTRA* extraversion, *HC* healthy controls, *IBD* inflammatory bowel disease, *MDD* major depressive disorder, *n/a* not applicable, *NEURO* neuroticism, *OPEN* openness, *PC* principal component, *PSOR* psoriasis, *PTSD* post-traumatic stress disorder, *RA* rheumatoid arthritis, *SCZ* schizophrenia spectrum disorders, *SD* standard deviation, *SMD* severe mental disorders (SCZ and BD), S*LE* systemic lupus erythematosus, *T1D* type 1 diabetes.

^a^Standardized values.

^b^*P* value of SMD vs HC: ANCOVA with adjustments for PC1, PC2, and genetic batch.

### Clinical assessment

Diagnostics are made by use of the Structured Clinical Interview (SCID-1) for the Diagnostic and Statistical Manual of Mental Disorders (DSM)-IV. Diagnostic interviews are performed by psychologists and physicians supervised by a clinical professor in psychiatry. The research personnel is all comprehensively trained for the interviews with a UCLA training program [48].

### Polygenic risk scores

DNA was extracted from blood and saliva samples collected at the time of inclusion of the participants. Samples were genotyped at deCODE Genetics (Reykjavik, Iceland) using Illumina Human OmniExpress-12, OmniExpress-24, and Global Screening Array chips. Pre-imputation quality control was performed using PLINK 1.9. Briefly, variants were excluded if they had a low genotyping rate (<95%), deviated from Hardy–Weinberg equilibrium (*P* < 10^−4^), or occurred at significantly different frequencies in different genotyping batches (FDR < 0.5). Whole-individual genotypes were excluded if they had low coverage (<80%), a high likelihood of contamination (heterozygosity above mean + 5 standard deviations) or mismatch between genetic and reported sex. The quality-controlled genotypes were phased using Eagle [49], and missing variants were then imputed with MaCH [50, 51] using haplotype reference consortium (HRC) trans-ethnic reference panel (version 1.1) [52]. Following the quality control and imputation procedure, variants with information score <0.8 or minor allele frequency <1% were removed. In addition, individual genotypes imputed with less than 75% confidence were set to missing, and the remaining ones were converted to best guess hard allelic dosages. PRS were computed following the method described by Purcell et al. [53] using PRSice-2 [54].

Our study included PRS of phenotypes with detected genetic associations to SMD and immune links, including (1) the autoimmune diseases rheumatoid arthritis (PRS-RA) [55], systemic lupus erythematosus (PRS-SLE) [56], inflammatory bowel disease (PRS-IBD) [57], psoriasis (PRS-PSOR) [58], celiac disease (PRS-CD) [59], type 1 diabetes (PRS-T1D) [60], as well as C-reactive protein (PRS-CRP) [61], (2) the mental disorders major depressive disorder (PRS-MDD) [62], anxiety (PRS-ANX) [63], autism spectrum disorders (PRS-ASD) [64], attention-deficit hyperactivity disorder (PRS-ADHD) [65], post-traumatic stress disorder (PRS-PTSD) [66], (3) the cognitive traits general intelligence (PRS-COG) [67] and the cognitive ability proxy educational attainment (PRS-EA) [68], and (4) the personality traits neuroticism (PRS-NEURO), openness (PRS-OPEN) and extraversion (PRS-EXTRA) [30]. We used the *P* value threshold of 0.05 for the selection of single-nucleotide polymorphisms (SNPs) in the PRS analyses. All PRS were standardized before analysis. See Supplementary Table 1 for a summary of the GWASs used for the calculation of PRS in our study.

### Immune markers

Immune markers were selected based on previously documented associations to SMD or neuropsychiatric symptomatology [14, 69–71], comprising interleukin-1 receptor antagonist (IL-1Ra), soluble interleukin-2 receptor (sIL-2R), interleukin-18 (IL-18), soluble glycoprotein 130 (sgp130), a proliferation-inducing ligand (APRIL), tumor necrosis factor receptor 1 (TNFR-1) and intercellular cell adhesion molecule 1 (ICAM-1). Following the same methods as described by Mørch et al. [43], plasma levels of the immune markers were measured in duplicate by enzyme immunoassays (EIA) applying commercially available antibodies (R&D Systems, Minneapolis, MN, USA) in a 384 format using a combination of a SELMA (Jena, Germany) pipetting robot and a BioTek (Winooski, VT, USA) dispenser/washer. Absorption was read at 450 nm with wavelength correction set to 540 nm using an ELISA plate reader (Bio-Rad, Hercules, CA, USA). Intra- and inter-assay coefficients of variation were <10% for all EIAs. For the immunoassays, blood was sampled between 8:00 and 17:00 using EDTA vials and the plasma was isolated within the next working day and stored at −80 °C. Plasma collection was performed subsequently as participants were included in the study, and “freezer storage time” (years) was recorded and included as a continuous independent variable in the main statistical model.

### Statistical analyses

All statistical analyses were performed using the Statistical Package for the Social Sciences (SPSS Inc., Chicago II, version 26). Histograms, Q–Q plots and Kolmogorov–Smirnov statistics were used to assess distributions of data. For group comparison of demographic and clinical data, we used *t* test and ANOVA for continuous variables and Chi-Square test for categorical variables. Pearson’s r or Spearman’s rho were used for correlation analyses. The main analyses were performed with Analysis of Covariance (ANCOVA), with immune markers as dependent variables. Immune marker distributions were carefully assessed, and all markers were logarithmically transformed with standardized removal of residuals more than 3× interquartile range (IQR) or 1.5× IQR below and above the first and third quartile, respectively, depending on the degree of deviation (3 IQR for gp130, sTNFR-1, IL-18, and ICAM-1 and 1.5 IQR for IL-1Ra, sIL-2R, and APRIL). First, we identified PRS and immune marker variables with indicated (*P* ≤ 0.1) differences between SMD and HC (Table 2 and Supplementary Table 2) and bivariate correlations (in SMD, Supplementary Table 3). We then performed the main analyses with these variables comparing immune marker differences between SMD and HC (“diagnosis variable”) with and without adjustments for PRS to assess the effect of PRS on immune marker differences. Moreover, all PRS correlating with immune markers in SMD were tested for interaction effects with the diagnosis variable. Main analyses, including interactions, were repeated in subsamples by substituting SMD with SCZ (“subsample SCZ-HC”) and BD (“subsample BD-HC”), respectively. Main analyses were adjusted for age at blood sampling, sex, freezer storage time, genetic principal components 1 and 2 and genetic batch by including these as independent variables in the ANCOVA. Based on established genetic overlap with SMD and immune links of SMD-related phenotypes, we applied a moderate correction of the significance level of *P* = 0.0125 (0.05/4) to correct for testing of PRS of the four groups of autoimmune disorders, mental disorders, cognitive traits, and personality traits.

## Results

### Demographic data and immune markers

The mean age was lower in SMD than in HC (*P* = 0.04) with younger participants in SCZ than in the BD and HC (both *P* < 0.001). The proportion of females was lowest in SCZ and highest in BD (all *P* < 0.001), see Table 1. Plasma levels of all immune markers differed significantly between SMD and HC (Supplementary Table 2); however, case-control differences are more specifically reported in other papers from our group [43–47] [submitted]. Bivariate correlation analyses of PRS and immune marker levels in SMD indicated three correlations with PRS of autoimmune diseases (“PRS-Autoimm”) and seventeen for PRS of mental disorders, traits, or cognitive abilities (“PRS-Ment”). Of these, there were one PRS-Autoimm (i.e., PRS-PSOR) and seven PRS-Ment (i.e., PRS-ANX, PRS-MDD, PRS-ADHD, PRS-PTSD, PRS-OPEN, PRS-COG, and PRS-EA) with the indicated difference between SMD and HC. Details are found in Table 2 and Supplementary Table 3.

### Immune marker levels: main effects of PRS

*PRS-Autoimm*: PRS-PSOR was nominally positively associated with plasma levels of sgp130 (*P* = 0.02); the plasma-level difference between SMD and HC decreased by 0.2 percentage points when adding PRS-PSOR to the model.

*PRS-Ment*: PRS-ANX was nominally negatively associated with sIL-2R (*P* = 0.02) and positively with IL-18 (*P* = 0.03); the plasma-level difference between SMD and HC of sIL-2R and IL-18 increased by 0.6 percentage points and decreased by 2.0 percentage points, respectively, when adding PRS-ANX to the models. PRS-EA was negatively associated with IL-1Ra (*P* = 0.01) and IL-18 (*P* = 0.01), and at nominal level with sIL-2R (*P* = 0.03); the plasma-level difference of IL-1Ra (18.6%) and sIL-2R (13.2%) were unchanged when adding PRS-EA to the models, whereas the plasma-level difference of IL-18 decreased by 0.7 percentage points. See Table 3 for statistical details of PRS main effects. There were no other significant main effects of PRS in the main analyses.

**Table 3:** Main effects of polygenic risk scores on immune markers^a^.

	Immune marker	df	F-ratio	*P* value	η_p_^2^	PP change^c^
Total sample						
PRS-PSOR	sgp130	1,1118	5.86	0.02	0.005	−0.2
PRS-ANX	sIL-2R	1,1050	5.19	0.02	0.005	0.6
	IL-18	1,1520	4.77	0.03	0.003	−2.0
PRS-EA	IL-1Ra	1,1087	6.46	0.01	0.006	0.0
	sIL-2R	1,1050	4.79	0.03	0.005	0.0
	IL-18	1,1520	6.93	0.01	0.005	−0.7
Subsample SCZ-HC^b^						
PRS-PSOR	sgp130	1,885	4.95	0.03	0.006	−0.2
PRS-ANX	IL-18	1,1196	3.92	0.05	0.003	−2.1
PRS-EA	IL-1Ra	1,861	4.09	0.04	0.005	0.4
	IL-18	1,1196	5.89	0.02	0.005	−0.4
Subsample BD–HC^b^						
PRS-EA	IL-1Ra	1,709	7.27	0.01	0.010	−0.2
	IL-18	1,1065	5.20	0.02	0.005	−0.6

*ANX* anxiety, *BD* bipolar spectrum disorders, *df* degrees freedom, *EA* educational attainment, *IL-18* interleukin-18, *IL-1Ra* interleukin-1 receptor antagonist, *η*_*p*_^*2*^ partial eta squared, *PP* percentage points, *PRS* polygenic risk score, *PSOR* psoriasis, *SCZ* schizophrenia spectrum disorders, *sIL-2R* soluble interleukin-2 receptor, *sgp130* soluble glycoprotein 130.

^a^Independent variables: PRS, age, sex, freezer storage time, patients vs healthy controls, genetic principal components 1 and 2 and genetic batch; dependent variables: immune markers.

^b^Subsample SCZ-HC: participants with schizophrenia spectrum disorders and healthy controls; subsample BD–HC: participants with bipolar spectrum disorders and healthy controls.

^c^Percentage points change in immune marker plasma-level difference between diagnostic subgroups and healthy controls by adding PRS to the statistical model.

### Immune marker levels: interaction effects of PRS

*PRS-Autoimm*: There were no significant interaction effects between PRS and the diagnosis variable.

*PRS-Ment*: Nominal SMD specific negative associations was found for PRS-EXTRA and IL-1Ra (*P* = 0.02 [interaction effect, *P* = 0.002]) and for PRS-OPEN and IL-1Ra (*P* = 0.02 [interaction effect, *P* = 0.02]) and sTNFR-1 (*P* = 0.04 [interaction effect, *P* = 0.04]). See Supplementary Fig. 1. There were no other significant interaction effects between PRS and the diagnosis variable.

### Subsample analysis

For separate analyses of SCZ-HC and BD–HC subsamples, see Table 3 and Supplementary Results. In general, subsample findings corresponded with the main findings. The association between PRS-EA and IL-1Ra in the BD–HC subsample survived correction for multiple testing (*P* = 0.01) and in addition there was a SCZ-specific association for PRS-EA and ICAM-1 (*P* = 0.003, [interaction effect, *P* = 0.01]). Levene’s test was significant in ANCOVA analysis of sTNFR-1 and in several analyses of IL-18.

## Discussion

The main findings of the study were significant associations of PRS-EA, and nominal associations for PRS-PSOR and PRS-ANX, with immune marker levels across patients with SMD and HC. Nominal associations that were specific for patients of immune levels and PRS-EXTRA and PRS-OPEN were detected by interaction analysis. Despite the limited explanatory power of PRS, the large sample allowed well-adjusted analyses indicating associations of genetic risk for autoimmune diseases and in particular for mental phenotypes with components of low-grade inflammation and immune activation in SMD; however, the correlations constituted small changes in immune marker differences between patients and HC. The involvement of several PRS-Ment might indicate effects on immune activation related to specific phenotypic features within SMD.

PRS-EA was negatively associated with IL-1Ra and IL-18 across SMD and HC, and both associations were confirmed in the SCZ-HC and BD–HC subsamples. We used educational attainment as a proxy for cognitive ability based on known associations [72], including genetic correlation [73]. Importantly, the clinical relevance of our findings is substantiated by reports of cognitive correlations in patient groups. In particular, elevated IL-1Ra is found to be associated with poorer cognition in BD [74] and IL-18 is associated with cognition although with mixed directions in Alzheimer’s disease and SCZ [75, 76]. Moreover, as IL-1Ra is regarded as a marker of IL-1b activity, the combined effects of IL-1Ra and IL-18 could implicate the involvement of Nod-like receptor (NLR) family, pyrin domain-containing 3 (NLRP3) inflammasomes, the major source of both IL-1 and IL-18. Interestingly, NLRP3 inflammasomes have recently been suggested to link psychological stress, depression, and systemic illness [77] and have been linked to severe mental illness [78]. An interaction effect in the subsample analysis suggested a SCZ-specific negative relationship of PRS-EA with ICAM-1 (Supplementary Results). Increased ICAM-1 with decreased general cognitive abilities is found in older people [79]. Similarly, a previous PRS study support of a role for cell adhesion molecules (CAMs) in cognitive function in SCZ [80]. Thus, our findings indicate a polygenic interplay regulating cytokines involved in the cognitive symptom dimension of SMD, potentially explaining immune abnormalities in subgroups with cognitive impairments.

In general, there were few associations with PRS-Autoimm and the examined inflammatory markers. However, our findings suggest an association between PRS-PSOR and sgp130 abnormalities in SMD. Psoriasis is an autoimmune disease with modest systemic aberrations of IL-6, CRP, TNF-α, E-selection, and ICAM [81] and comorbidity with SCZ [82]. Gp130 is the common signal transducer in the IL-6 cytokine family and reflect the activation of several of these cytokines. The positive correlation is also supported by elevated expression of gp130 in patients with psoriasis [83]. Although a minor impact on the difference between SMD and HC, the current association of sgp130 might reflect the genetic risk of sgp130 abnormalities in autoimmune-related subgroups of SMD, in line with the broad comorbidity of autoimmune diseases with SMD [17]. The trend-level lower PRS-PSOR in SMD versus HC is at odds with a recently indicated small positive genetic correlation between SCZ and psoriasis [84]. The difference might be related to the current use of another and more recent GWAS of psoriasis. However, the finding suggests a potential of leveraging genetics of autoimmune diseases in elucidating immune mechanisms in SMD with the increasing power of GWAS.

Associations of PRS-ANX with IL-18 and sIL-2R were also indicated. Anxiety is genetically correlated to [3] and a common comorbidity of SCZ. The comorbidity exists also in prodromal stages [85], suggesting associations beyond overt psychotic symptoms. PRS-ANX was positively associated with IL-18 across SMD and HC, as well as in SCZ. The positive link with IL-18 is further supported by elevated levels of IL-18 in anxiety including increased levels with severity, indicating clinical implications [86]. While PRS-ANX was negatively associated with sIL-2R across SMD and HC, subgroup analyses also suggested a BD-specific negative association not detected in the overall sample. Others have reported decreased levels of sIL-2R in anxiety [87], thus supporting the current negative correlation. However, sIL-2R may vary with clinical state in BD [88]. Thus, the interaction between PRS-ANX and BD suggest an interplay with other susceptibilities in sIL-2R regulation related to clinical characteristics. Moreover, the positive association of PRS-ANX with IL-18 and a negative association with sIL-2R as robust marker of T-cell activation could implicate that innate and adaptive immunity may differently affect anxiety.

In addition to the main effects of PRS of autoimmune and SMD-related mental phenotypes, PRS of personality traits were related to immune markers, particularly IL-1Ra, in interactions with SMD. The findings are in line with the documented association of personality traits with immune aberrations [38]. Decreased PRS-OPEN and PRS-EXTRA were both associated with increased IL-1Ra in SMD and SCZ, similar to the correlation between PRS-OPEN and sTNFR-1 in SMD. Interestingly, both openness and SCZ may be linked to dopamine activity in substantia nigra [89] and creativity [90] and a negative association with psychotic experiences was recently shown in a non-clinical sample [91]. The effects correspond with evidence suggesting widespread interactions in immune mechanisms [92]. Although speculative, one might hypothesize a mechanism involving the hypothalamic–pituitary–adrenal (HPA) axis given its association with immune responses and dysregulation in SMD [93, 94]. The interactions support complex associations underlying immune mechanisms also in psychiatric disorders, in line with previous studies [95]. Moreover, common immune pathways linking personality traits and SMD symptomatology may be hypothesized.

The strengths of this study are large, well-characterized sample of participants and a selection of immune markers robustly associated with SMD. The inclusion of SMD-related mental PRS in combination with the immune markers is to our knowledge the first of its kind. Limitations include a cross-sectional format that makes us unable to assess the stability of the associations. Also, the repeated testing increases risk of type 1 error; however, the analyses were based on known immune differences between SMD and HC and all PRS phenotypes were selected based on genetic associations to SMD and associations to immune markers. Moreover, given the lack of studies of underlying mechanisms of systemic inflammation in SMD, a stricter correction for multiple testing may lead to ignoring hypotheses that merit further investigation. Still, the moderate correction for multiple testing and significant Levene’s test of some of the main ANCOVAs, warrants cautious interpretation of the results. Moreover, plasma was isolated and frozen within the next working day, as immediate freezing was not feasible. Although conflicting evidence [96] studies suggest reasonable stability of immune markers under various conditions [97]. Thus, while bias from potential blood handling issues or other non-specific effects cannot be fully excluded, a significant impact on the results seems less likely in the current setting of well-adjusted analyses and a large sample size. Moreover, major influence by unidentified factors is further prevented by the careful removal of outliers from the immune markers. The main associations explained small amounts of the difference in immune marker plasma level between SMD and HC. This is in line with findings in comparable studies [40]. The limited explanatory power is not unexpected given the complexity of SMD mechanisms, the impact of non-genetic influences on the immune system including stress responses related to clinical state [98], high level of polygenicity with potential interaction effects and missing heritability of the phenotypes [68, 99] and uncertain heritability of immune components [100].

In conclusion, we found associations of PRS of immune-linked SMD-related mental phenotypes and nominally of psoriasis with immune markers in SMD and HC. This suggests that genetic susceptibility of these phenotypes might be involved in mechanisms underlying systemic immune abnormalities often seen in SMD, which seems to be partly disease-specific. Although representing small changes, our findings indicate genetic susceptibilities in SMD immune dysregulation for further investigation, potentially of specific interest in subgroups based on autoimmune or mental characteristics, which could be of future relevance in personalized intervention studies. Moreover, the involvement of IL-1Ra and IL-18 in the detected association with various PRS may underscore a particular role for innate immunity in SMD.

## Supplementary information

Supplementary table 1

Supplementary table 2

Supplementary table 3

Supplementary results

Supplementary figure 1
