# Supplementary material for: Immune marker levels in severe mental disorders: associations with polygenic risk scores of related mental phenotypes and psoriasis
Source: Transl Psychiatry. 2022 Jan 26;12:38. doi: 10.1038/s41398-022-01811-6 (PMC8792001; doi:10.1038/s41398-022-01811-6)
Supplement: Supplementary file 1 — Supplementary table 1 [file 41398_2022_1811_MOESM1_ESM.docx]

**Supplementary table 1.** Summary of the GWASs used for calculation of PRS

| **Phenotype** | **Phenotype abbreviation** | **Quantitative or qualitative trait** | **Year of publication** | **Publication URL** | **Title of the publication** | **First or last author** |
| --- | --- | --- | --- | --- | --- | --- |
| Celiac disease | CD | Case/control | 2010 | <https://www.nature.com/articles/ng.543> | Multiple common variants for celiac disease influencing immune gene expression | Dubois PC |
| Inflammatory Bowel Disease | IBD | Case/control | 2017 | <https://www.ncbi.nlm.nih.gov/pubmed/28067908> | Genome-wide association study implicates immune activation of multiple integrin genes in inflammatory bowel disease | de Lange KM |
| Psoriasis | PSOR | Case/control | 2012 | <https://europepmc.org/abstract/pmc/pmc3322238> | Combined analysis of genome-wide association studies for Crohn disease and psoriasis identifies seven shared susceptibility loci | Ellinghaus D |
| Rheumatoid arthritis | RA | Case/control | 2014 | <http://www.ncbi.nlm.nih.gov/pubmed/24390342> | Genetics of rheumatoid arthritis contributes to biology and drug discovery | Okada Y |
| Systemic Lupus Erythematosus | SLE | Case/control | 2015 | <http://dx.doi.org/10.1038/ng.3434> | Genetic association analyses implicate aberrant regulation of innate and adaptive immunity genes in the pathogenesis of systemic lupus erythematosus | Bentham J |
| T1D | T1D | Case/control | 2009 | <https://www.nature.com/articles/ng.381> | Genome-wide association study and meta-analysis find that over 40 loci affect risk of type 1 diabetes | Barrett JC |
| Chronic Inflammation | CRP | Quantitative | 2018 | <https://www.sciencedirect.com/science/article/pii/S0002929718303203?via%3Dihub> | Genome Analyses of >200,000 Individuals Identify 58 Loci for Chronic Inflammation and Highlight Pathways that Link Inflammation and Complex Disorders | Symen Ligthart |
| Attention Deficit / Hyperactivity Disorder | ADHD | Case/control | 2017 | <https://www.nature.com/articles/s41588-018-0269-7> | Discovery Of The First Genome-Wide Significant Risk Loci For ADHD | Ditte Demontis |
| Anxiety | ANX | Case/control | 2020 | <https://www.nature.com/articles/s41380-019-0559-1> | A major role for common genetic variation in anxiety disorders | Purves et al |
| Autism Spectrum Disorder | ASD | Case/control | 2019 | <https://www.nature.com/articles/s41588-019-0344-8> | Identification of common genetic risk variants for autism spectrum disorder | Jakob Grove |
| Major depressive disorder | MDD | Case/control | 2018 | <https://www.nature.com/articles/s41588-018-0090-3> | Genome-wide association analyses identify 44 risk variants and refine the genetic architecture of major depressive disorder | Wray et al |
| Posttraumatic stress disorder | PTSD | Case/control | 2019 | <https://www.ncbi.nlm.nih.gov/pubmed/31594949> | International meta-analysis of PTSD genome-wide association studies identifies sex- and ancestry-specific genetic risk loci | Nievergelt CM |
| Intelligence | COG | Quantitative | 2018 | <https://www.nature.com/articles/s41588-018-0152-6> | Genome-wide association meta-analysis in 269,867 individuals identifies new genetic and functional links to intelligence | J. E. Savage |
| Educational attainment | EA | Quantitative | 2018 | <https://www.nature.com/articles/s41588-018-0147-3.epdf> | Gene discovery and polygenic prediction from a 1.1-million-person GWAS of educational attainment | Lee et al |
| Extraversion | EXTRA | Quantitative | 2016 | <http://www.nature.com/ng/journal/v49/n1/full/ng.3736.html?foxtrotcallback=true> | Genome-wide analyses for personality traits identify six genomic loci and show correlations with psychiatric disorders | Min-Tzu Lo |
| Neuroticism | NEUR | Quantitative | 2016 | <http://www.nature.com/ng/journal/v49/n1/full/ng.3736.html?foxtrotcallback=true> | Genome-wide analyses for personality traits identify six genomic loci and show correlations with psychiatric disorders | Min-Tzu Lo |
| Openness | OPEN | Quantitative | 2016 | <http://www.nature.com/ng/journal/v49/n1/full/ng.3736.html?foxtrotcallback=true> | Genome-wide analyses for personality traits identify six genomic loci and show correlations with psychiatric disorders | Min-Tzu Lo |
