# Supplementary material for: Immune marker levels in severe mental disorders: associations with polygenic risk scores of related mental phenotypes and psoriasis
Source: Transl Psychiatry. 2022 Jan 26;12:38. doi: 10.1038/s41398-022-01811-6 (PMC8792001; doi:10.1038/s41398-022-01811-6)
Supplement: Supplementary file 2 — Supplementary table 2 [file 41398_2022_1811_MOESM2_ESM.docx]

**Supplementary table 2**. Immune data

| Immune markers^a^, median (IQR) | SCZ | BD | HC | p-value^d^ | SMD vs HC |
| --- | --- | --- | --- | --- | --- |
| IL-1Ra^b^ | 241 (325) | 191 (274) | 197 (268) | 0.005 | SMD>HC |
| sIL-2R^c^ | 0.28 (0.2) | 0.26 (0.2) | 0.24 (0.1) | <0.001 | SMD>HC |
| sgp130^c^ | 215 (51) | 208 (59) | 224 (60) | <0.001 | HC>SMD |
| sTNFR-1^c^ | 1.75 (0.7) | 1.68 (0.5) | 1.60 (0.8) | 0.001 | SMD>HC |
| IL-18^b^ | 978 (1613) | 769 (1447) | 731 (1226) | <0.001 | SMD>HC |
| APRIL^b^ | 250 (217) | 253 (242) | 334 (272) | <0.001 | HC>SMD |
| ICAM-1^c^ | 271 (116) | 273 (107) | 251 (97) | <0.001 | SMD>HC |

^a^Reported in Engh et al., 2021; Mørch et al., 2017; Mørch et al., 2019; Szabo et al., 2022; Sheikh et al., submitted.

^b^pg/ml; ^c^ng/ml; ^d^p-value of SMD vs HC: ANCOVA for immune markers (log-transformed) with adjustments for age, sex and freezer storage time.

Abbreviations: APRIL = A proliferation-inducing ligand, BD = bipolar spectrum disorders, HC = healthy controls, ICAM-1 = Intercellular Adhesion Molecule 1, IL-1Ra = Interleukin-1 Receptor antagonist, IL-18 = Interleukin-18, IQR = interquartile range, SCZ = schizophrenia spectrum disorders, SMD = severe mental disorders (SCZ and BD), sgp130 = soluble glycoprotein 130, sIL-2R = soluble Interleukin-2 Receptor, sTNFR-1 = soluble Tumour Necrosis Factor 1.
