# Supplementary material for: Immune marker levels in severe mental disorders: associations with polygenic risk scores of related mental phenotypes and psoriasis
Source: Transl Psychiatry. 2022 Jan 26;12:38. doi: 10.1038/s41398-022-01811-6 (PMC8792001; doi:10.1038/s41398-022-01811-6)
Supplement: Supplementary file 3 — Supplementary table 3 [file 41398_2022_1811_MOESM3_ESM.docx]

**Supplementary table 3.** Bivariate correlations^a^ between PRS and immune markers

in all patients

|  |  | IL-1Ra | sIL-2R | sgp130 | sTNFR-1 | IL-18 | APRIL | ICAM-1 |
| --- | --- | --- | --- | --- | --- | --- | --- | --- |
| Autoimmune diseases and CRP | PRS-CD | -0.03 (0.41) | -0.02 (0.69) | 0.04 (0.35) | 0.003 (0.93) | -0.03 (0.39) | 0.06 (0.11) | -0.01 (0.78) |
|  | PRS-IBD | 0.01 (0.90) | -0.002 (0.96) | 0.01 (0.72) | -0.02 (0.66) | -0.01 (0.75) | -0.01 (0.79) | 0.04 (0.22) |
|  | PRS-PSOR | 0.06 (0.15) | -0.03 (0.43) | **0.09 (0.02)** | 0.04 (0.27) | -0.01 (0.83) | 0.05 (0.21) | 0.05 (0.12) |
|  | PRS-RA | -0.004 (0.92) | -0.04 (0.38) | -0.06 (0.17) | 0.01 (0.82) | 0.01 (0.85) | -0.004 (0.92) | -0.02 (0.63) |
|  | PRS-SLE | 0.06 (0.12) | 0.02 (0.63) | 0.04 (0.30) | 0.01 (0.85) | **0.08 (0.03**) | **0.08 (0.02)** | -0.01 (0.85) |
|  | PRS-T1D | 0.004 (0.92) | -0.03 (0.54) | -0.05 (0.20) | -0.03 (0.51) | -0.04 (0.26) | 0.03 (0.49) | -0.02 (0.51) |
|  | PRS-CRP | 0.03 (0.44) | -0.02 (0.57) | -0.01 (0.79) | -0.01 (0.73) | 0.02 (0.58) | 0.01 (0.72) | 0.02 (0.57) |
| Mental disorders | PRS-ADHD | 0.03 (0.41) | -0.01 (0.73) | 0.02 (0.60) | 0.05 (0.18) | 0.04 (0.25) | 0.03 (0.49) | 0.02 (0.67) |
|  | PRS-ANX | 0.05 (0.25) | **-0.11 (0.01)** | 0.04 (0.32) | 0.002 (0.97) | **0.09 (0.01)** | 0.02 (0.65) | -0.04 (0.22) |
|  | PRS-ASD | 0.05 (0.26) | **-0.08 (0.04)** | 0.02 (0.62) | 0.04 (0.37) | -0.01 (0.74) | -0.04 (0.28) | 0.04 (0.28) |
|  | PRS-MDD | **0.08 (0.04)** | -0.01 (0.77) | **0.07 (0.08)** | 0.02 (0.68) | 0.02 (0.51) | 0.03 (0.46) | 0.03 (0.39) |
|  | PRS-PTSD | **0.07 (0.07)** | 0.03 (0.43) | 0.01 (0.90) | 0.02 (0.58) | -0.003 (0.94) | 0.03 (0.36) | 0.01 (0.78) |
| Cogn traits | PRS-COG | **-0.08 (0.06)** | 0.03 (0.48) | 0.02 (0.62) | 0.03 (0.46) | -0.04 (0.29) | -0.03 (0.43) | -0.05 (0.18) |
|  | PRS-EA | **-0.07 (0.08)** | **-0.08 (0.06)** | -0.03 (0.50) | **-0.07 (0.07)** | **-0.08 (0.04)** | **-0.08 (0.04)** | **-0.09 (0.01)** |
| Personality traits | PRS-EXTRA | **-0.09 (0.03)** | 0.03 (0.44) | 0.01 (0.82) | 0.01 (0.75) | **-**0.01 (0.69) | -0.03 (0.39) | -0.04 (0.25) |
|  | PRS-NEURO | 0.02 (0.68) | -0.04 (0.31) | **-0.11 (0.01)** | 0.03 (0.42) | -0.01 (0.87) | -0.001 (0.98) | 0.02 (0.60) |
|  | PRS-OPEN | **-0.10 (0.02)** | -0.05 (0.20) | 0.03 (0.40) | **-0.09 (0.03)** | -0.04 (0.22) | -0.02 (0.55) | -0.02 (0.56) |

^a^The correlation coefficient (Pearson’s r except Spearman’s rho for PRS-SLE) and p-value in parenthesis is given, p ≤0.1 in bold

Abbreviations: ADHD = attention deficit hyperactivity disorder, AGREE = agreeableness, ANX = anxiety, APRIL = A proliferation-inducing ligand, ASD = autism spectrum disorder, CD = Crohn’s disease, COG = cognition, Cogn traits = cognitive traits, CRP = C-reactive protein, EA = educational attainment, EXTRA = extraversion, IBD = inflammatory bowel disease, IL-1Ra = Interleukin-1 Receptor antagonist, IL-18 = Interleukin-18, ICAM-1 = Intercellular Adhesion Molecule-1, MDD = major depressive disorder, NEURO = neuroticism, OPEN = openness, PSOR = psoriasis, PTSD = post-traumatic stress disorder, RA = rheumatoid arthritis, sIL-2R = soluble Interleukin-2 Receptor, sgp130 = soluble glycoprotein 130, sTNFR-1 = soluble Tumour Necrosis Factor-1, SLE = systemic lupus erythematosus, T1D = type 1 diabetes
