# Supplementary material for: Immune marker levels in severe mental disorders: associations with polygenic risk scores of related mental phenotypes and psoriasis
Source: Transl Psychiatry. 2022 Jan 26;12:38. doi: 10.1038/s41398-022-01811-6 (PMC8792001; doi:10.1038/s41398-022-01811-6)
Supplement: Supplementary file 4 — Supplementary results [file 41398_2022_1811_MOESM4_ESM.docx]

**Supplementary results.** Interaction effects of PRS in diagnostic subsamples

*Subsample SCZ-HC*: SCZ specific negative associations were found for PRS-EA and ICAM-1 (p = 0.003 [interaction effect, p = 0.01]) and PRS-EXTRA and IL-1Ra (p = 0.03 [interaction effect, p = 0.001]).

*Subsample BD-HC*: A BD specific negative association was found for PRS-ANX and sIL-2R (p = 0.01 [interaction effect, p = 0.04]).
