# Supplementary material for: Immune marker levels in severe mental disorders: associations with polygenic risk scores of related mental phenotypes and psoriasis
Source: Transl Psychiatry. 2022 Jan 26;12:38. doi: 10.1038/s41398-022-01811-6 (PMC8792001; doi:10.1038/s41398-022-01811-6)
Supplement: Supplementary file 5 — Supplementary figure 1 [file 41398_2022_1811_MOESM5_ESM.pdf]

Supplementary figure 1. Scatterplots of immune markers and polygenic risk scores based on interaction effects between PRS and diagnosis group (HC and SMD)

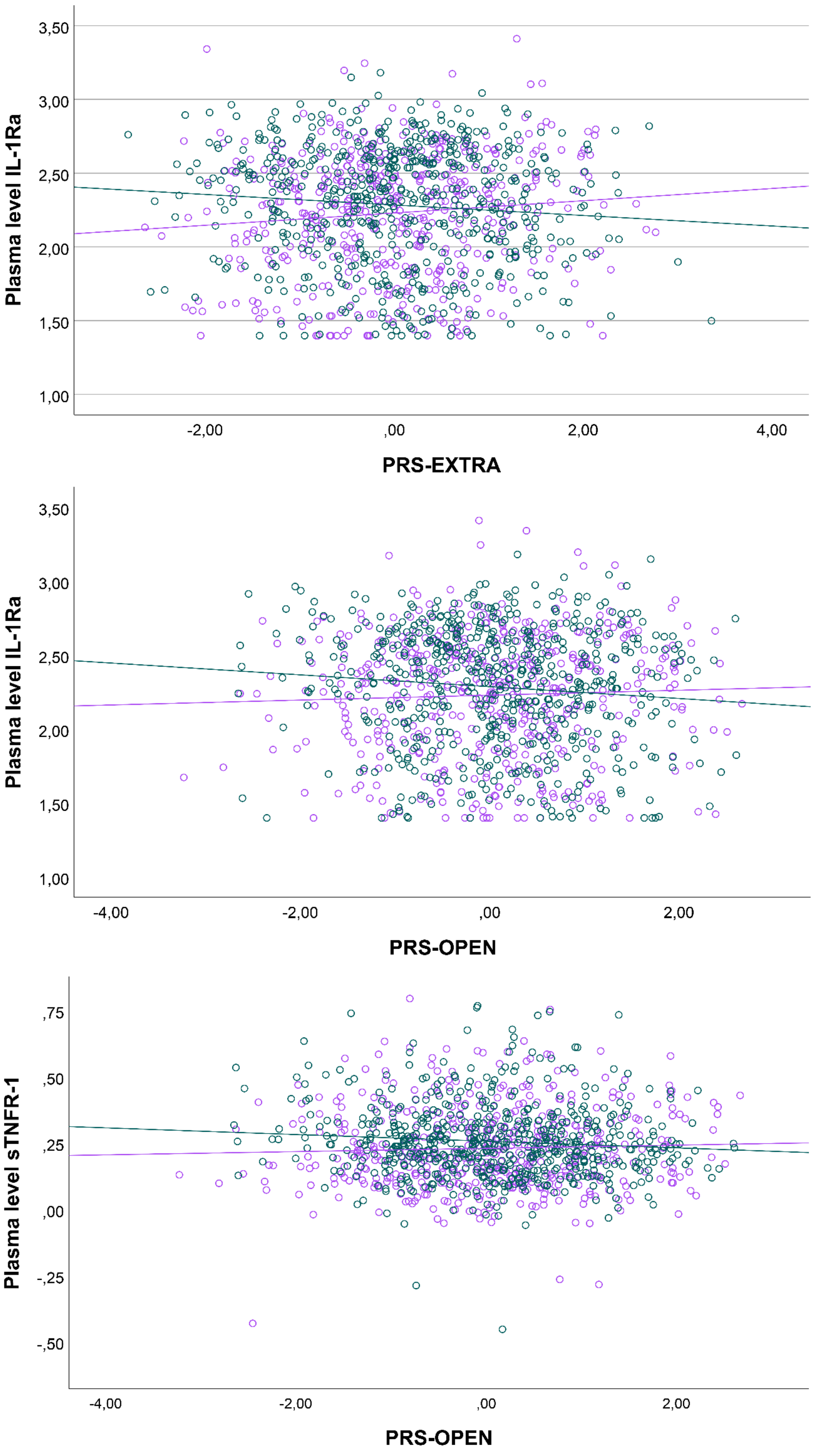

Abbreviations: EXTRA = extraversion, HC = healthy controls, IL-1Ra = Interleukin-1 Receptor antagonist, OPEN = openness, PRS = polygenic risk score, SMD = severe mental disorders, sTNFR-1 = soluble Tumour Necrosis Factor-1.
